# Microbial (co)infections: Powerful immune influencers

**DOI:** 10.1371/journal.ppat.1010212

**Published:** 2022-02-03

**Authors:** Ali Hassan, Nicolas Blanchard

**Affiliations:** Toulouse Institute for Infectious and Inflammatory Diseases, Infinity, University of Toulouse, CNRS, Inserm, UPS, Toulouse, France; Universitat Zurich, SWITZERLAND

## Abstract

It is well established that by modulating various immune functions, host infection may alter the course of concomitant inflammatory diseases, of both infectious and autoimmune etiologies. Beyond the major impact of commensal microbiota on the immune status, host exposure to viral, bacterial, and/or parasitic microorganisms also dramatically influences inflammatory diseases in the host, in a beneficial or harmful manner. Moreover, by modifying pathogen control and host tolerance to tissue damage, a coinfection can profoundly affect the development of a concomitant infectious disease. Here, we review the diverse mechanisms that underlie the impact of (co)infections on inflammatory disorders. We discuss epidemiological studies in the context of the hygiene hypothesis and shed light on the sometimes dual impact of germ exposure on human susceptibility to inflammatory disease. We then summarize the immunomodulatory mechanisms at play, which can involve pleiotropic effects of immune players and discuss the possibility to harness pathogen-derived compounds to the host benefit.

## Introduction

The main functions of our immune system are to provide defenses against invasion by pathogens and tumor cells and to promote tissue homeostasis and repair. Through the process of immune tolerance, the immune system can distinguish self and nonself so that an immune response develops against nonself elements, while no harm is inflicted upon self. The disruption of tolerance may lead to the development of autoimmune diseases, which manifest by an attack on self-tissues as if they were foreign.

The efficiency of the immune system to defend against pathogens, but also the severity of its attacks against self after tolerance breakdown, are largely affected by intrinsic (for example, genetics) and extrinsic factors (for example, environmental cues or exposome), including exposure to pathogenic and/or commensal microorganisms. It is now well established that by modulating various immune functions, host infection may alter the course of concomitant inflammatory diseases, of both infectious and autoimmune etiologies. In fact, infections can either ameliorate or aggravate the clinical outcome of an inflammatory disorder. Numerous studies have contributed to identify the variety of mechanisms that govern the immune modulation by microorganisms during a defined pathology. In some cases, with helminths in particular, some of the molecules and pathways discovered are even being clinically tested as new therapeutic strategies against specific autoimmune diseases.

In this review, we aim to provide a comprehensive overview of the major mechanisms by which exposure to infectious agents throughout the host lifetime shapes the immune status.

### Hygiene hypothesis: Is germ exposure always beneficial for the host?

In 1989, Strachan proposed for the first time the hygiene hypothesis for allergic diseases based on the fact that hay fever was less common in children with older siblings [1]. He reasoned that older children might have been less frequently exposed to microorganisms compared to their younger siblings and proposed that microbial exposure in early life could later protect against hypersensitivities. This hypothesis was supported by several epidemiological studies and has been extended not only to other allergic but also to autoimmune diseases. In the past few decades, the incidence of autoimmune and allergic diseases, such as asthma, atopic dermatitis, type 1 diabetes (T1D) and multiple sclerosis (MS) has indeed increased in more industrialized compared to less industrialized countries [2,3]. While several factors such as genetics, exposure to sun and vitamin D, and socioeconomic levels may in part explain this increase, a strong correlation with the decreased incidence of infectious diseases has been noted. For example, Sotgiu and colleagues reported a correlation between the increase of MS incidence and the eradication of malaria in Sardinia [4]. A lower risk to develop MS and T1D has also been linked to early exposure to a diverse microbial community. For example, naturally helminth-infected and/or treated MS patients showed less exacerbation and fewer magnetic resonance imaging changes compared to uninfected and placebo patients, respectively [5,6]. In addition, a study on a large cohort in Finland showed that children who spent their childhood with an indoor dog, which is thought to increase the probability of exposure to germs, had a reduced chance of developing T1D compared to children without an indoor dog [7]. In Gabon and Vietnam, 2 independent studies have found that schoolchildren infected with *Schistosoma* or *Ascaris* nematodes presented lower levels of allergen reactivity compared to their uninfected classmates [8,9]. These observations were supported by the finding that anthelminthic treatment of infected children resulted in an increased atopic reactivity [10]. A case–control study performed in Japan on 4,000 patients showed that the frequency of infection by *Strongyloides stercoralis*, a nematode parasite that infects the lungs and intestine, was lower in the group of patients with autoimmune liver diseases than in the control group [11]. Elsewhere, children reported to have taken antibiotics during infancy had a greater risk to develop inflammatory bowel disease [12], asthma, eczema, and hay fever later in life [13]. Taken together, these studies highlight a positive correlation between early exposure to microbes and reduced risk of autoimmune/allergic disorders.

Besides these beneficial effects, it has also been documented that exposure to microbes could induce or aggravate autoimmune/allergic disorders. Infection with Epstein–Barr virus (EBV) correlates with the development of systemic lupus erythematosus (SLE) and MS, as SLE and MS patients display higher seroprevalence of EBV compared to healthy controls [14,15] and some autoreactive CD4^+^ T cell clones can cross-react with EBV peptides [16]. Moreover, different types of pathogens like *Acinetobacter* and *Pseudomonas aeruginosa* (*P*. *aeruginosa*) have been associated with the induction of MS [17]. Several viruses have been correlated with T1D. For example, Coxsackie virus B4 can be detected in pancreatic but also intestinal biopsies of patients with T1D, especially after a recent onset of the disease [18].

Lastly, it has also become clear that coinfection can profoundly impact the clinical outcome of a concomitant infectious disease. A recent emblematic example is the *Leishmania* parasite-borne RNA virus (LRV) that infects and replicates within certain isolates of *Leishmania* parasites and has been correlated with treatment failure and metastasis of *Leishmania* lesions in patients [19,20]. Beyond LRV, EBV/malaria coinfection in children from sub-Saharan Africa can either cause an exacerbation of malaria or the development of EBV-related pathologies [21]. Moreover, a sero-epidemiological study among Tanzanian infants showed that those who developed severe malaria by 1 year of age were more likely to have been seropositive for filarial antigen earlier in life [22]. However, since the filarial and plasmodial parasites can share the same mosquito vector, a limitation of this work is that filarial seroreactivity may just be the sign of a higher risk of exposure to *Plasmodium*, independently from a direct impact of filarial parasites on malaria severity. In contrast, a study of Senegalese children showed that those who were mildly infected with *Schistosoma haematobium* had a lower density of *Plasmodium falciparum* compared to non-coinfected children [23]. In line with this observation, coinfection with *Ascaris lumbricoides* was associated with a reduction in cerebral malaria risk [24]. These differences may be explained by the nature of the helminth parasites and the age of children, as younger children may respond differently to coinfection.

In summary, multiple lines of evidence indicate that exposure to viruses, parasites, or bacteria can positively or negatively regulate the clinical outcome of concomitant autoimmune or infectious diseases. We will now discuss the major immune modulatory mechanisms that underlie this phenomenon.

### Immunomodulation through type I interferons: Antiviral cytokines with high immunomodulatory potential

Almost all cells can produce type I interferons (IFN-I) and express their receptor. IFN-I play a pivotal role in antiviral defense, but since these cytokines have diverse effects on a wide variety of immune cells, they can differently modulate many pathologies. In the case of murine cutaneous leishmaniasis, copresence of LRV worsens the severity and metastasis of skin lesions by inducing the production of proinflammatory cytokines and chemokines such as IL-6, TNF-α, CCL5, CXCL10, and IFN-I via the activation of the TLR3-TRIF pathway in macrophages [25]. Interestingly, not only LRV but also coinfection with exogenous, nonparasite-borne viruses like lymphocytic choriomeningitis virus (LCMV) and Toscana virus increase *Leishmania* pathology due to IFN-I production in this mouse model. One of the effects of IFN-I is to down-regulate the expression of IFN-γ receptor that is normally required for *Leishmania* parasite control [26]. A similar detrimental role of IFN-I was recently reported during experimental *P*. *aeruginosa* infection. In this case, a filamentous bacteriophage produced by the bacteria exacerbates *P*. *aeruginosa–*mediated pathology through the induction of IFN-I via the TLR3-TRIF pathway, causing a reduction in TNF-α expression and altered bacterial phagocytosis by macrophages [27]. In addition to their impact on parasitic and bacterial infections, viral infections can change the prognosis of fungal diseases. A recent study in human macrophages has reported a virus-mediated enhancement of vomocytosis (a nonlytic extrusion leading to the expulsion of engulfed microbes by phagocytes) of the fungus *Cryptococcus neoformans* in an IFN-I–dependent manner [28].

**Fig 1:**
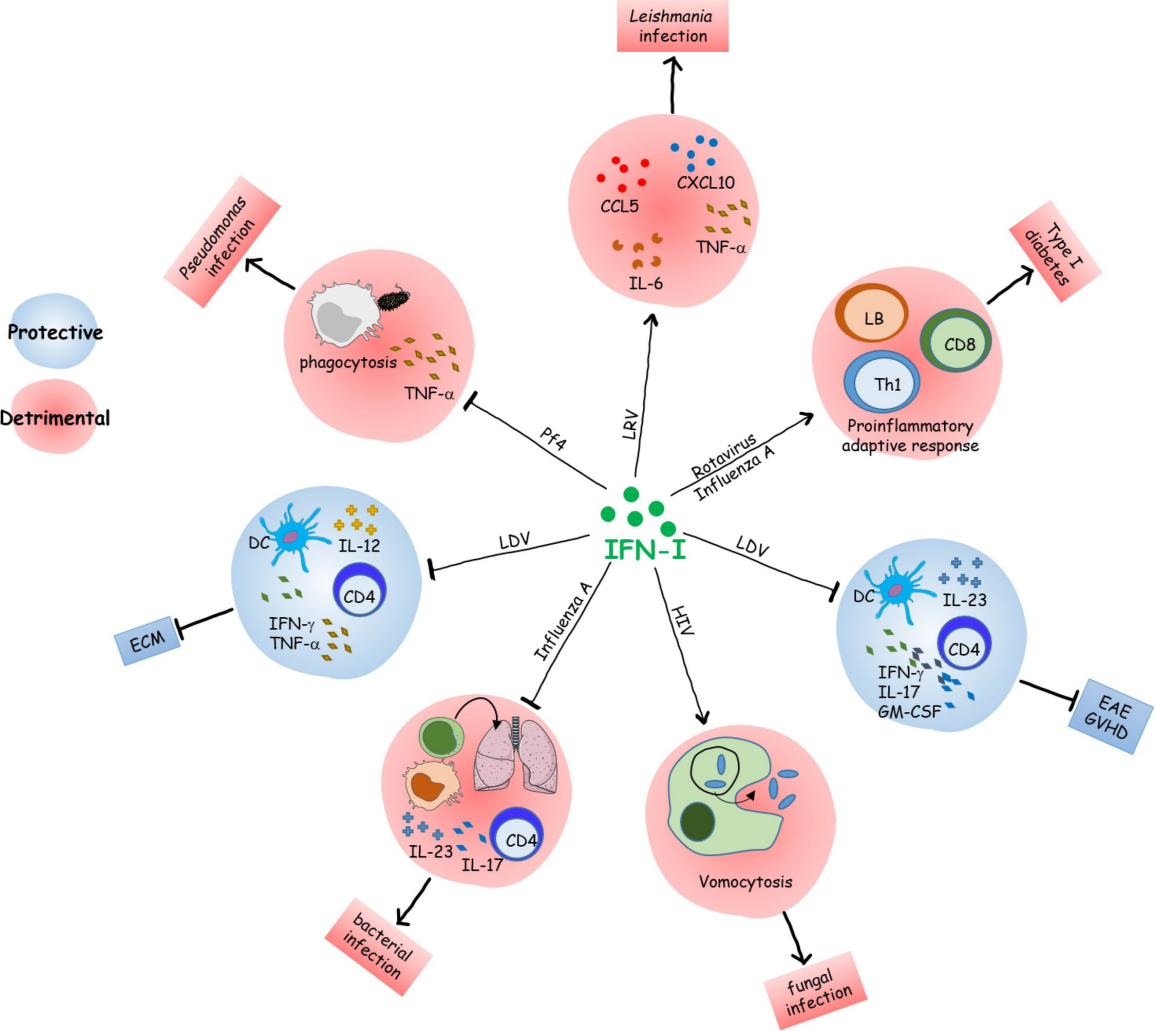
Functional modulation of immune cells by infection-mediated IFN-I. Several immune functions can be regulated negatively or positively by IFN-I produced during concomitant pathologies, resulting in protective or detrimental phenotypes. DC, dendritic cell; EAE, experimental autoimmune encephalomyelitis; ECM, experimental cerebral malaria; GVHD, graft versus host disease; IFN-I, type I interferon; LDV, lactate dehydrogenase-elevating virus; LRV, *Leishmania* parasite-borne RNA virus.

Another example illustrating the role of IFN-I is the infection by influenza (flu) virus, which affects millions of people around the world each year. Flu infection is often associated with secondary respiratory bacterial infections, which can dramatically worsen the clinical outcome of susceptible patients. Several studies in animal models have shown that previous or simultaneous influenza A virus infection enhances susceptibility to gram-negative and gram-positive bacterial pneumonia and that the pathology is promoted by IFN-I signaling. Mechanistically, IFN-I inhibits macrophage and neutrophil recruitment to the lung by altering the production of several chemoattractants like CXCL1 and CXCL2 through transcriptional and epigenetic modulation [29,30]. In addition, IFN-I suppresses type 17 immune responses by decreasing IL-17 and IL-23 production, and ROR-γt expression [31,32]. The deleterious impact of IFN-I was also observed on dendritic cells (DCs). In fact, concurrent pulmonary infection with influenza A virus is associated with an IFN-I–mediated decrease in MHC-I and MHC-II expression on DC, resulting in reduced activation of CD4^+^ and CD8^+^ T cells, and impaired clearance of Mycobacteria [33]. Such immune impairment could explain why a clinical study reported the reactivation of tuberculosis in some patients undergoing IFN-I treatment for chronic viral hepatitis [34]. Altogether, these results indicate that IFN-I can act negatively on a large scale by modulating a variety of innate and adaptive immune cells (**Fig 1**).

Conversely, in some situations, the immunomodulatory effects of IFN-I can play a protective role for the host. For example, coinfection with lactate dehydrogenase-elevating virus (LDV) protects mice against *Plasmodium berghei* ANKA-mediated experimental cerebral malaria, a vascular pathology that is typically caused by CD8^+^ effector T cells and is sustained by CD4^+^ Th1 responses. In this case, LDV induces the production of IFN-I, which promotes a decrease in the number of DC and in their capacity to produce IL-12, and consequently in their ability to induce Th1 responses. The critical role of IFN-I was confirmed as *Ifnar*1 KO mice display normal DC number and functions upon LDV infection [35]. These data are in line with the previous observations that (i) IFN-I elicits DC death by inducing the expression of certain proapoptotic proteins [36] and that (ii) IFN-I can regulate, in a dose-dependent manner, the transcription of IL-12p40 in DC by modifying the phosphorylation of STAT molecules [37].

IFN-I triggered by infection can also modulate the outcome of autoimmune disorders. Pane and colleagues suggested that rotavirus infection accelerates diabetes in nonobese diabetic (NOD) mice by inducing IFN-I release by plasmacytoid DC (pDC) through the TLR7/MDA5 pathways. Secreted IFN-I induces the activation of B and T cells, including autoreactive CD8^+^ T cells [38]. Accordingly, several studies have linked respiratory virus-induced IFN-I to T1D aggravation. For example, influenza A infection induces IFN-I production by pDC in mice and patients, which is associated with Th1-mediated T1D development [39,40]. While IFN-I seems to play an important role, other factors such as virus replication and cytolytic effects could contribute to T1D exacerbation [41]. Conversely, LDV infection protects mice against experimental autoimmune encephalomyelitis (EAE), a murine model of MS. In fact, LDV infection impairs IL-12 and IL-23 expression by DC through IFN-I signaling, leading to altered development of autoreactive CD4^+^ T cells [35]. In addition to this qualitative defect, LDV-derived IFN-I reduces the number of DC and T cells and is protective in a model of graft versus host disease [42].

Of note, the timing of IFN-I signaling is a key factor in the outcome of protective versus detrimental responses. Reports have suggested that early IFN-I production can elicit a protective response against *Mycobacterium tuberculosis (Mtb)* infection. In contrast, late IFN-I signaling suppresses the host-protective immune response [43]. Along this line, a retrospective study of Coronavirus Disease 2019 (COVID-19) patients found that early IFN-I treatment improves disease outcome, whereas late IFN-I administration is associated with increased mortality [44]. Based on these findings, we speculate that the timing of infection by the IFN-I–inducing pathogen should critically determine the outcome of the second pathology.

**Fig 2:**
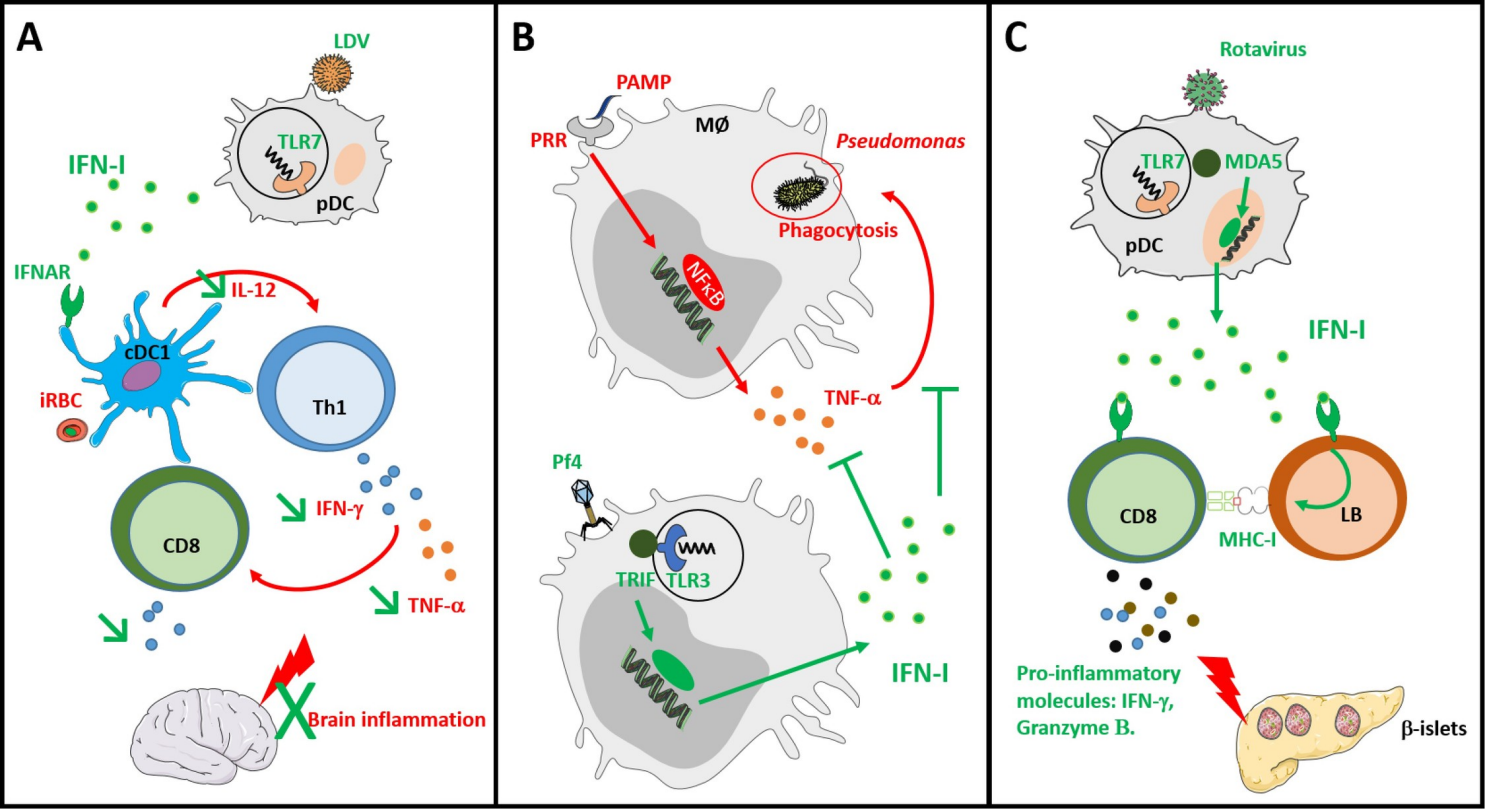
Examples of immunomodulation through infection-mediated IFN-I. IFN-I display high modulatory potential as illustrated by their capacity to positively or negatively modulate infectious or autoimmune diseases and to shape the functions of innate and adaptive immune cells. **(A)** DCs present parasitic antigens to T cells, which induces cell migration to the brain and proinflammatory cytokine production causing cerebral malaria. LDV infection induces IFN-I production by pDC. Released IFN-I causes a quantitative and qualitative defect in DC and, consequently, a decreased inflammatory response leading to the protection against cerebral malaria. **(B)** Bacterial ligands stimulate NF-κB translocation leading to TNF-α production. TNF stimulates bacterial phagocytosis by macrophages. Phage RNA triggers IFN-I production through TLR3-mediated TRIF signaling. IFN-I inhibits TNF production and bacterial phagocytosis and prevents infection clearance. **(C)** Rotavirus RNA induces IFN-I by pDC through TLR7/MDA5 signaling. IFN-I induces lymphocyte activation, B cells up-regulate MHC-I expression and present autoantigens to CD8+ T cells, which, in turn, produce proinflammatory molecules and cause death of pancreatic β cells. (IFN-I production and modulatory functions are presented in green). DC, dendritic cell; IFN-I, type I interferon; LDV, lactate dehydrogenase-elevating virus; pDC, plasmacytoid DC; TNF, tumor necrosis factor.

In conclusion, IFN-I has diverse, sometimes opposed, effects on immune and cellular responses (**Fig 2**). A moderate level of IFN-I is important to initiate an optimal immune response, while higher levels can be deleterious. Consequently, the type of pathogen, its capacity to induce low/high amounts of IFN-I, and in case of a secondary infection whether it occurs at the peak or resolution of IFN-I signaling are critical determinants of the fate of the immune response and the pathology.

### Immunomodulation through tolerance to tissue damage and regulatory immunity

#### Tolerance to tissue damage (or disease tolerance)

During an infection, the host may undergo tissue damage directly caused by pathogen toxicity or by an inadequately resolved inflammatory response. Accordingly, a mechanism of tolerance is employed as a defense strategy to limit the negative impact of different forms of stress, thereby minimizing tissue damage. Failure to establish this tolerance can lead to a dramatic change in the clinical outcome of secondary infections, independently from pathogen burden. One example is the lethal coinfection of influenza virus and *Legionella pneumophila*. Interestingly, the use of attenuated bacteria, or of mice lacking the immune components induced during coinfection, like neutrophils, natural killer (NK) cells, or T and B cells (Rag2 KO), does not rescue them from mortality. Instead, mortality is associated with lung epithelial damage and a downregulation of genes involved in tissue repair. In this context, treatment with an epithelial growth factor contributing to tissue homeostasis and development increases survival [45]. This pioneer study revealed the impact of the loss of tolerance on infection-induced tissue damage and the importance of tissue repair for the clinical outcome of secondary infection. Similarly, selective inhibition of the membrane-tethered matrix metalloprotease MT1-MMP protects the tissue from damage and is correlated with a better clinical outcome during flu/*Streptococcus pneumoniae* mouse coinfection, without altering the immune response or cytokine expression [46].

#### Regulatory response in the context of coinfection

In addition to the immune-independent mechanisms of disease tolerance and tissue repair described above, regulatory immune responses are also critical processes that promote tissue repair and restore homeostasis. A hallmark cell type is the regulatory T cell (Treg), which, in addition to its immunosuppressive functions, plays an important role in tissue repair [47,48]. Interestingly, treatment with amphiregulin, a growth factor expressed by Treg, ameliorates survival during experimental influenza/*L*. *pneumophila* coinfection [45]. Yet, overall, the role of Treg and their regenerative properties during coinfection remains poorly understood. The role of immunosuppressive cytokines in controlling disease progression is well characterized. IL-10 is a hallmark suppressive cytokine, and multiple examples illustrate its regulatory functions. In a study on macaques and mice, *Plasmodium* infection attenuates intestinal inflammation caused by *Salmonella typhimurium* in an IL-10–dependent manner. This improvement is linked to a decrease in the expression of IL-12, IFN-γ, IL-17A, and in CXCL1-mediated neutrophil infiltration in the cecum, due to *Plasmodium*-induced IL-10 [49]. Although IL-10–mediated suppression of proinflammatory responses is beneficial in certain cases, it can also be detrimental. For example, ongoing, or previously resolved, helminth infections were reported to impair the efficacy of anti-influenza vaccination in various mouse studies. Helminth infection reduces the neutralizing capacity of influenza-specific antibodies via the expansion of CD49b+ LAG3+ Tr1 cells expressing IL-10 [50]. Moreover, helminth-mediated IL-10 expression was shown to dampen IFN-γ expression by CD8^+^ T cells, which exacerbates *Toxoplasma gondii* (*T*. *gondii*) infection [51]. Mechanistically, IL-10 can directly restrict CD8^+^ T cell activation and function by modifying cell surface glycosylation, which increases the antigenic threshold required for T cell activation [52]. Along the same line, blocking IL-10R or neutralizing IL-10 restores Th1 (IFN-γ, TNF-α) and Th17 (IL-17A, IL-17F) responses and ameliorates *Mtb* control that is blunted, respectively, by influenza A and helminth infection [53,54]. In contrast, deworming of helminth-coinfected patients is correlated with a significant decline of IL-10 level, without improvement of tuberculosis [55], showing that, in reality, coinfections involve a complex network of pro and anti-inflammatory cytokines, and likely other mechanisms beyond immunosuppression.

#### Regulatory response in the context of infection autoimmunity

Regarding the innate immune compartment, a recent study has shown that gamma herpesvirus protects against house dust mite–induced experimental asthma by promoting the replacement of embryonic resident alveolar macrophages by bone marrow–derived regulatory monocytes, which colonize the lungs and alter the ability of DC to trigger a specific Th2 response [56]. This indicates that some viruses could be protective through remodeling the immune microenvironment toward a more regulatory profile.

Concerning the role of regulatory adaptive cells, it was shown that helminth parasite–infected MS patients show a better disease outcome associated with an increase in CD4^+^ CD25^+^ Foxp3^+^ Treg cell frequency, IL-10 and TGF-β production, and a decrease in IL-12 and IFN-γ–secreting cells compared to noninfected patients [5,6,57]. A parasite modulation of the Smad7/TGF-β axis was proposed as a possible explanation. The protective role of Treg was observed in mice in a study where the clinical evolution of EAE was ameliorated by *Plasmodium chabaudi* infection [58], and adoptive transfer of *Plasmodium*-conditioned DC or CD4^+^ Treg was sufficient to ameliorate EAE through the suppression of autoreactive CD4^+^ T cell response [58,59]. In NOD mice, helminth parasites as well as their products prevent diabetes via the induction of CD4^+^ Treg and the production of IL-10 [60,61]. Mechanistically, parasite eggs induce Treg development in a TGF-β–dependent manner through the induction of TGF-β–activating α8 integrin, LAP, and galectin 1 and 3 expression on CD4^+^ T cells, in addition to the expression of IL-2 and IL-10 by DC [60]. However, IL-10–deficient NOD mice are protected against diabetes [62]. Moreover, IL-2 treatment reverses established disease in NOD mice by acting on local Treg but independently from IL-10 and TGF-β expression [63]. Thus, parasite-induced protection of NOD mice seems to be mediated by regulatory mechanisms that rely on Treg but are partially independent from IL-10 production. Finally, helminth-induced Treg modulate airway inflammation and inhibit asthma, by reducing antigen-specific immunoglobulin E (IgE) and pulmonary eosinophilia in mouse models of asthma [64,65]. Interestingly, protective pulmonary Treg are induced by helminth-activated CD1d ^high^ regulatory B cells (Breg) via IL-10 production [66,67]. A similar mechanism was found in several autoimmune experimental models such as lupus [68], arthritis [69], and EAE [70]. In the context of allergy, a clinical study on African children reported that IL-10 is induced by chronic schistosomiasis and that it is inversely correlated with reactivity in a house dust mite skin test, suggesting that IL-10 plays a central role in the helminth-mediated suppression of atopy [8].

**Fig 3:**
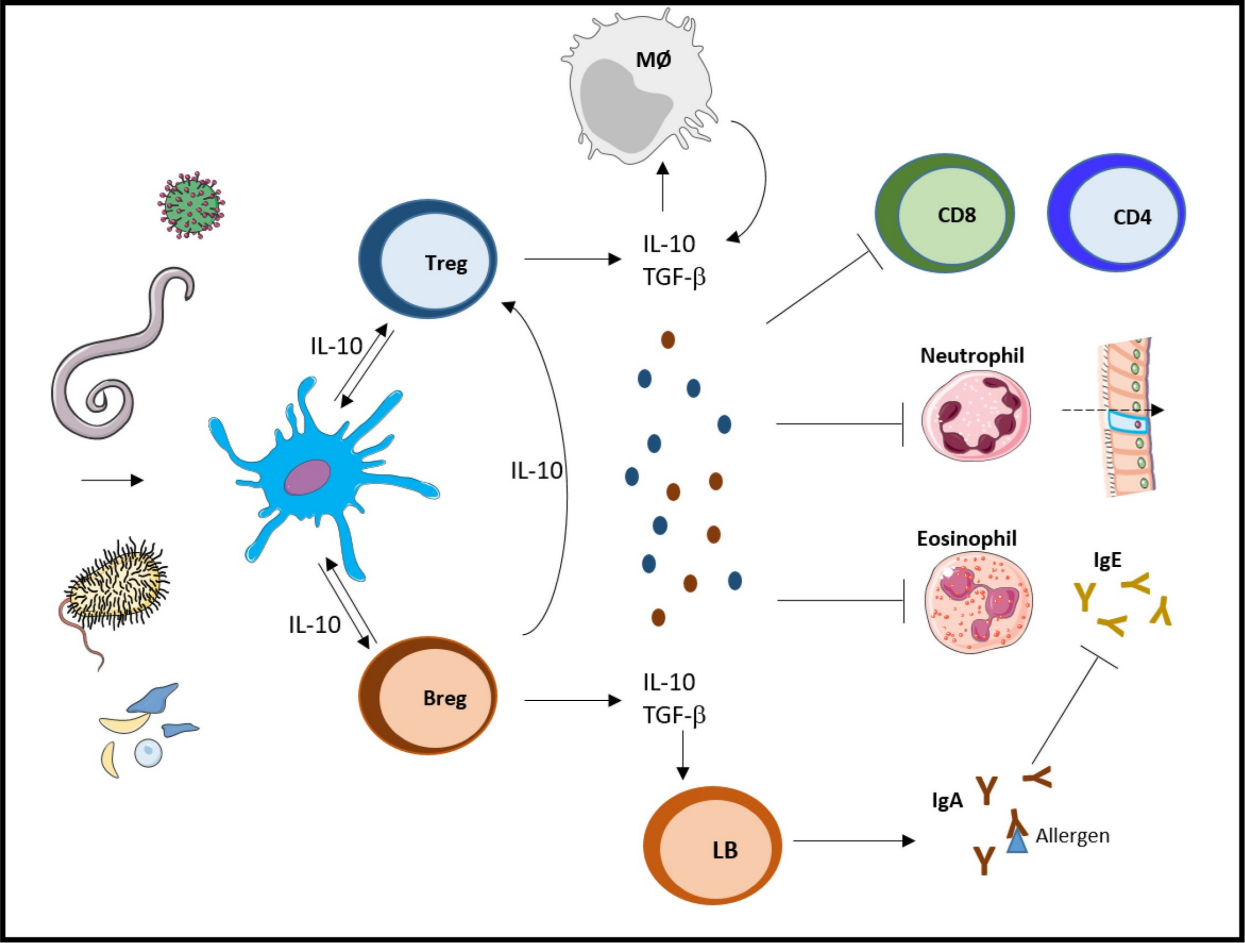
Immune response modulation by pathogen-activated regulatory T and B cells. Virus, bacteria, parasite, or pathogen-derived molecules could induce the development of a regulatory immune response by activating lymphocytes or DC, which stimulate Treg or Breg via the production of IL-10. Together, regulatory cells produce immunomodulatory cytokines like IL-10 and TGF-β, which inhibit CD4^+^ and CD8^+^ T cell differentiation and functions, chemokine-mediated neutrophil infiltration, eosinophil inflammatory response, and stimulate B cells to produce modulatory IgA, which neutralize allergen and control IgE. Consequently, the clinical outcome of a concomitant disease could be impacted positively or negatively by regulatory T and B cells, depending on the nature of the pathology. Breg, regulatory B cell; DC, dendritic cell; IgA, immunoglobulin A, IgE, immunoglobulin E; IL-10, interleukin 10; TGF-β, transforming growth factor beta; Treg, regulatory T cell.

In summary, infection-induced Treg and Breg are often simultaneously useful in controlling inflammation, in large part through IL-10, but they may also have IL-10–independent functions and nonredundant roles (**Fig 3**).

### Immunomodulation through type 1/type 2 immunity switching

Depending on the class of pathogen and the tissue microenvironment, activated immune cells can polarize toward phenotypically and functionally distinct cell populations, leading to the establishment of immune responses of different types, type 1 and 2 being the most common. Type 1 immunity relates to an inflammatory cytotoxic response mounted to fight intracellular pathogens and cancer cells. Type 2 immunity relates to responses that are critical for host resistance against helminthic infections, and it comprises immunosuppressive and tissue repair functions. Switching between type 1 and type 2 immunity can drastically change the way by which the immune system reacts to a specific pathological context and, thus, the course of disease. In humans and mice, *Mtb* elicits a proinflammatory type 1 response. Hence, patients coinfected with helminths, a strong driver of Th2 responses, develop a more protracted and severe tuberculosis [71,72]. Helminth coinfection leads to a decrease in Th1, Th17, and NK cell frequency, an increase in Th2 and Treg, and a modified profile of pro/anti-inflammatory cytokine production [53,71,73]. Unexpectedly, profiling of CD4^+^ T cells in Kenyan individuals showed that *Schistosoma* coinfection does not impair *Mtb*-specific Th1 cytokine production. In fact, coinfected individuals had a higher frequency of *Mtb*-specific CD4^+^ T cells expressing IFN-γ than *Mtb*-monoinfected individuals [74]. Differences in the stages of worm lifecycle, which can drive Th2 as well as Th1 responses, may explain these discordant results. The order of infection, a difficult parameter to define in humans, may also be involved. This hypothesis is supported by several murine coinfection studies. When *T*. *gondii* infection precedes helminth infection, a decrease in the expression of the transcription factor GATA3, in the production of IL-4, IL-13, as well as an increase in the production of IFN-γ by CD4^+^ T cells is observed [75]. In contrast, when helminth infection precedes *T*. *gondii* infection, mice display a defective IFN-γ and IL-12 production by CD8^+^ T cells and cDC1, correlated with an increased IL-4 and IL-10 production leading to an enhanced *T*. *gondii* cyst load in the brain of coinfected mice [51,76].

Beyond T cell responses, switching of macrophage polarization can also modulate the outcome of coinfection. For example, a helminth-promoted switch from M1 to alternatively activated macrophages via IL-4 signaling leads to decreased expression of several proinflammatory cytokines and chemokines such as IL-1β, TNF-α, IL-6, IL-12, CCL(1-2-4-11), and CXCL(2-9-10), and is associated with tuberculosis exacerbation [72,77–79]. In addition to the classical mechanisms of action (pro or anti-inflammatory response), a recent in vitro study shed light on an original mechanism by which functionally modulated macrophages could aggravate infection. *Mtb* infection was shown to increase HIV dissemination via the induction of permissive immunomodulatory macrophages and the formation, through an IL-10/STAT3 signaling pathway, of intercellular nanotubes, which facilitate HIV transmission between cells [80].

In the context of autoimmunity, MS patients treated with anthelminthic molecules show an increased clinical MS activity correlated with higher IFN-γ and IL-12 expression and a drop in the number of regulatory cells [57]. Conversely, in mice, helminth infection tends to reduce EAE severity by curtailing IFN-γ, IL-17, and IL-12 expression and enhancing IL-4 production via STAT6 signaling [57,81]. The same protective effect is observed in a murine model of rheumatoid arthritis where a preestablished *Schistosoma* infection causes a downregulation of the level of anticollagen IgG, IFN-γ, IL-17A, TNF-α, IL-1β, and IL-6 and an upregulation of IL-4 [82].

Altogether, these studies show that infection can modulate concomitant disease by switching the type of immune response. The lapse before disease induction is critical for determining the phenotype of the induced immune response and, consequently, the clinical outcome.

### Immunomodulation through chemokine regulation and immune cell migration

By modulating the migration of immune cells to the local site of infection or inflammation, an infection can drastically affect the clinical evolution of the concomitant pathology. Teo and colleagues have shown that Chikungunya virus (CHIKV) coinfection protects mice against cerebral malaria by altering parasite-specific CD8^+^ T cells trafficking to the brain. In coinfected mice, splenic IFN-γ–mediated CXCL9 and CXCL10 production induces retention of CXCR3-expressing pathogenic CD8^+^ T cells in the spleen and prevents their migration to the brain [83]. Conversely, preinfection of mice with *Plasmodium* abolishes CHIKV-induced joint swelling due, in part, to altered migration of pathogenic CD4^+^ T cells to joints. Altered migration is partially dependent on CXCR3, in addition to an increased CD4^+^ T cell apoptosis in lymph nodes [84]. A lower CD8^+^ T cell infiltration in the brain was also reported during *Schistosoma*/*Plasmodium* coinfection. However, in this case, as IFN-γ expression is decreased, the lower brain infiltration is not due to an accumulation of IFN-γ–mediated chemokine in the spleen but rather to a *Schistosoma*-mediated lack of CD8^+^ T cell development and a limited impairment of the blood–brain barrier [85]. In another experimental study, *Schistosoma* infection disrupts the control of *Helicobacter pylori* colonization by inducing a misdirection of *H*. *pylori*-experienced CXCR3^+^ Th1 cells that fail to home to the stomach [86].

In addition to T cells, neutrophil migration is a process that is critical in the control of infections and may be affected by a coinfection. A preestablished gut-restricted helminth infection protects against *P*. *aeruginosa* coinfection by increasing neutrophil recruitment to the mouse lungs [87]. Mechanistically, helminth-induced IL-4 and IL-13 increase the transcription and activity of the lipoxygenase gene *Alox15* [87], leading to the generation of the inflammatory lipid mediator hepoxilin A3 [88], which drives the migration of neutrophils across the mucosal epithelial barrier into the airspace [89].

During autoimmunity, LCMV infection in mice prevents T1D by stimulating an IFN-γ–mediated CXCL10 production, which attracts islet-infiltrated T cells back from the islets to the pancreatic draining lymph node [90]. By contrast, chemokine production plays a detrimental role during influenza A infection by inducing T cell, monocyte, and neutrophil trafficking to the brain, which causes the development of autoimmune encephalomyelitis in autoimmune-prone T-cell receptor transgenic 2D2 mice. RNA-seq analysis showed that several chemokines are upregulated in the central nervous system (CNS) of infected mice [91]. At the molecular level, binding of the flu viral RNA to TLR7 activates the MyD88/NF-κB pathway, which, in turn, enhances cell recruitment to the CNS [91]. Thus, the balance of chemokine production between lymphoid organs and the site of pathology, which is affected by the nature of the pathogen and the type of infection (systemic or local), is a key factor to determine cell migration. Targeting chemokine production could be helpful for ameliorating the clinical outcome of certain pathologies.

### Modulation of humoral immunity

Perhaps less studied than cellular immunity, the modulation of humoral immunity also represents a mechanism by which an infection may impact concomitant diseases. In sub-Saharan Africa, infants are often coinfected with gamma herpesvirus, which infects B cells and is proposed to partially underlie the very slow acquisition of immunity to severe malaria in children. Using a mouse model of coinfection, Matar and colleagues have shown that acute, but not latent, gamma herpesvirus infection suppresses the antimalarial humoral response. In fact, coinfected mice are defective in generating malaria-specific IgG producing plasma cells. Interestingly, a viral protein causes a defect in germinal center maintenance by reducing the ability of B cells to communicate with follicular helper T cells (Tfh), probably by inducing the expression of the suppressive ligand PD-L1 [92]. Similar mechanisms were observed in mice sequentially infected with flu and *S*. *pneumoniae*. This coinfection results in a deadly phenotype and a reduced level of virus-specific IgG, IgM, and IgA, lower numbers of B and plasma cells, altered Tfh responses, and germinal center maintenance [93].

In the context of autoimmunity, through molecular mimicry and bystander activation, infections may lead to immune tolerance breakdown and cause the activation of B cells and the production of autoantibodies, leading to the development of autoimmune disorders [14,94]. Human studies have suggested that *P*. *falciparum* infection is correlated with an increased level of autoreactive IgG that may recognize antigens derived from brain tissue [95]. Interestingly, a more recent study in mouse malaria has shown that *Plasmodium* DNA, via synergistic activation of TLR9/IFN-γR, induces the development of a special population of “atypical” B cells, which are T-bet^+^ CD11c^+^ and produce anti-erythrocyte antibodies, causing the development of autoimmune anemia [96]. It remains to be established if the same B cell population could be the origin of the brain autoreactive IgG in cerebral malaria patients.

In the future, detailed analyses of the mechanisms of pathogen-mediated suppression or activation/differentiation of B and Tfh should be helpful to identify new target pathways and therapeutic molecules.

### Immunomodulation through trained immunity

**Fig 4:**
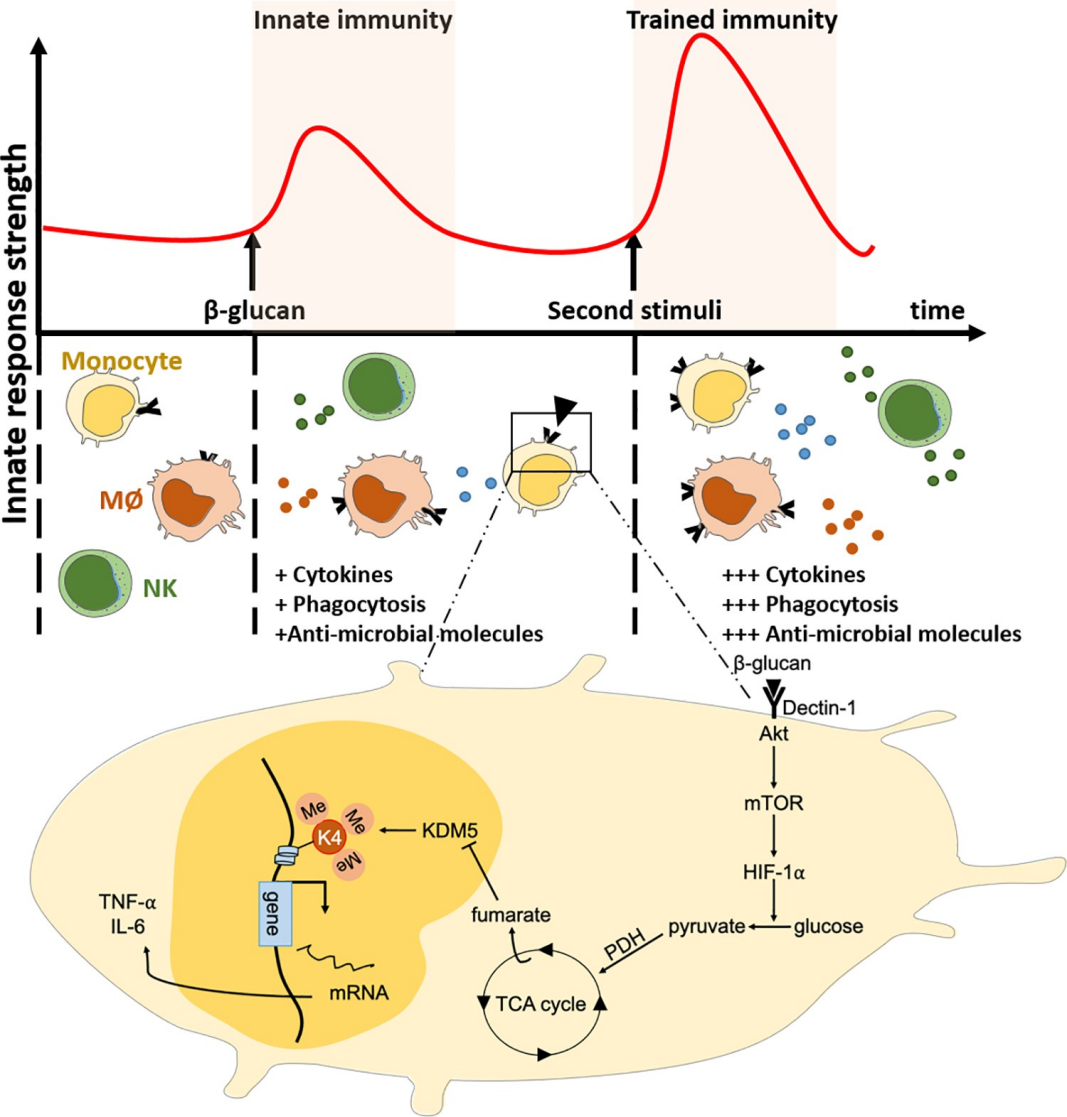
Infection-induced trained immunity. Upon encounter with a pathogen and sensing of its specific ligands (for example, β-glucan), innate immune responses become activated to orchestrate immune defense. In addition, this first microbial stimulus leads to the development of a relatively long-term, metabolically and epigenetically controlled, innate memory response called trained immunity. Ligand recognition triggers a series of intracellular cascades that activates several metabolic pathways such as glycolysis and TCA. Certain metabolites derived from these processes such as fumarate can modulate enzymes involved in epigenetic remodeling such as the histone demethylase KDM5, leading to the modulation of histone methylation status of innate immune genes. Following a second stimulation with the same, or with an unrelated pathogen, the trained innate cells are able to respond more strongly and to control the infection more efficiently. HIF-1α, hypoxia-inducible factor 1α; IL-6, interleukin 6; KDM5, lysine-specific demethylase 5; mTOR, mechanistic target of rapamycin; NK, natural killer cell; PDH, pyruvate dehydrogenase; TCA, tricarboxylic acid; TNF-ɑ, tumor necrosis factor alpha.

Conventionally, specific antigenic memory is the hallmark of adaptive immune T and B cells. Another type of nonspecific memory mediated by innate cells, called “trained immunity,” has been recently discovered [97] (**Fig 4**). Trained immunity is based on the functional reprogramming of innate immune cells through immunological, metabolic, and epigenetic modifications that occur upon encounter with certain pathogen-associated molecular patterns, and result in stronger immunity to a second infection by the same or distinct (cross-protection) pathogens, and last for a relatively long time (i.e., a few months and up to 1 year or longer in certain cases). One of the well-documented examples is trained immunity elicited by *Candida albicans* and the fungal cell wall component β-glucan. Exposure to β-glucan, or to a low dose of *C*. *albicans*, affords protection against lethal fungal reinfection in mice lacking functional T and B cells. Interestingly, preexposed monocytes display a functional reprogramming and produce more IL-6 and TNF-α in response not only to *C*. *albicans*, but also to other pattern recognition receptor ligands and bacterial stimulations [98]. Mechanistically, trained monocytes display an epigenetic activating change in the H3K4me3 profile of the related gene promoters through a dectin-1 signaling pathway [98,99]. Moreover, β-glucan–induced trained immunity confers protection against secondary nonfungal infection by modulating IL-32 production and increasing inflammatory and antimicrobial responses through a mechanism requiring monocytes/macrophages as well as IL-1 signaling [100,101]. Likewise, human PBMC exposed to *Plasmodium*-infected red blood cells show an increased production of IL-6 and TNF-α correlated with epigenetic remodeling, in response to secondary TLR stimulation. Interestingly, *Plasmodium*-infected Kenyan children present the same epigenetic modifications even after antimalaria treatment [102]. Thus, epigenetic changes may, at least partially, explain why the majority of multiply infected individuals are asymptomatic. In addition to natural infection, trained immunity is induced by vaccination. For example, antituberculosis vaccination is protective against Mycobacteria as well as against nontargeted pathogens [103,104]. Arts and colleagues reported that bacille Calmette-Guérin (BCG) vaccination promotes protection against yellow fever viremia. Protection is mediated by an epigenetic remodeling of monocytes, through Nod2 activation [103], which drives IL-1β and TNF-α production [105] and in turn contributes to enhance adaptive T cell responses [104]. Importantly, trained immunity is thought to be induced in the bone marrow. In fact, infectious challenge promotes myelopoiesis and educates hematopoietic stem cells to generate trained innate cells [106,107]. This observation is essential to help understand the duration of trained immunity considering the relatively fast turnover of monocytes. Consequently, the use of trained stem cells as a new type of nonspecific vaccination is enticing and under active scrutiny. During the COVID-19 pandemic, correlative data suggest that countries that never implemented a universal BCG vaccination policy display higher rate of mortality and reported cases of SARS-CoV2 infection, indicating that BCG may offer protection against the virus [108]. Overall, these studies highlight the novel concept of microbe-mediated nonspecific vaccines.

In several contexts of autoimmune diseases, monocytes from patients display features consistent with a trained immunity phenotype, like increased cytokine production, and metabolic and epigenetic modifications. For example, during SLE and rheumatoid arthritis, isolated human monocytes display increased production of IL-1β, IL-6, and TNF-α [109,110], activated PI3K/mTOR and MAPK signaling pathways [111], high expression of CD80, CD86, and HLA-DR [112], suggesting a better capacity of presenting antigens to T cells, including the presentation of self-antigens to autoreactive T cells. The potentialization of the proinflammatory responses triggered by trained immunity supports the notion that training likely plays a deleterious role in the context of autoimmune diseases. Conversely, in a murine model of autoimmunity, injection of helminth extracts induces alternatively activated macrophages with an anti-inflammatory profile, correlated with the abrogation of the adaptive response and resistance to EAE [113]. However, a direct impact of helminth extracts on adaptive cells cannot be excluded. Overall, studies have started to reveal an association between autoimmunity in humans and a trained immunity phenotype; however, no direct causal link has been formally proven yet.

Currently, trained immunity stands as an appealing target to improve treatment and vaccination by inhibiting or activating the metabolic, epigenetic, and immune pathways involved in the innate training process.

## Conclusions

**Fig 5:**
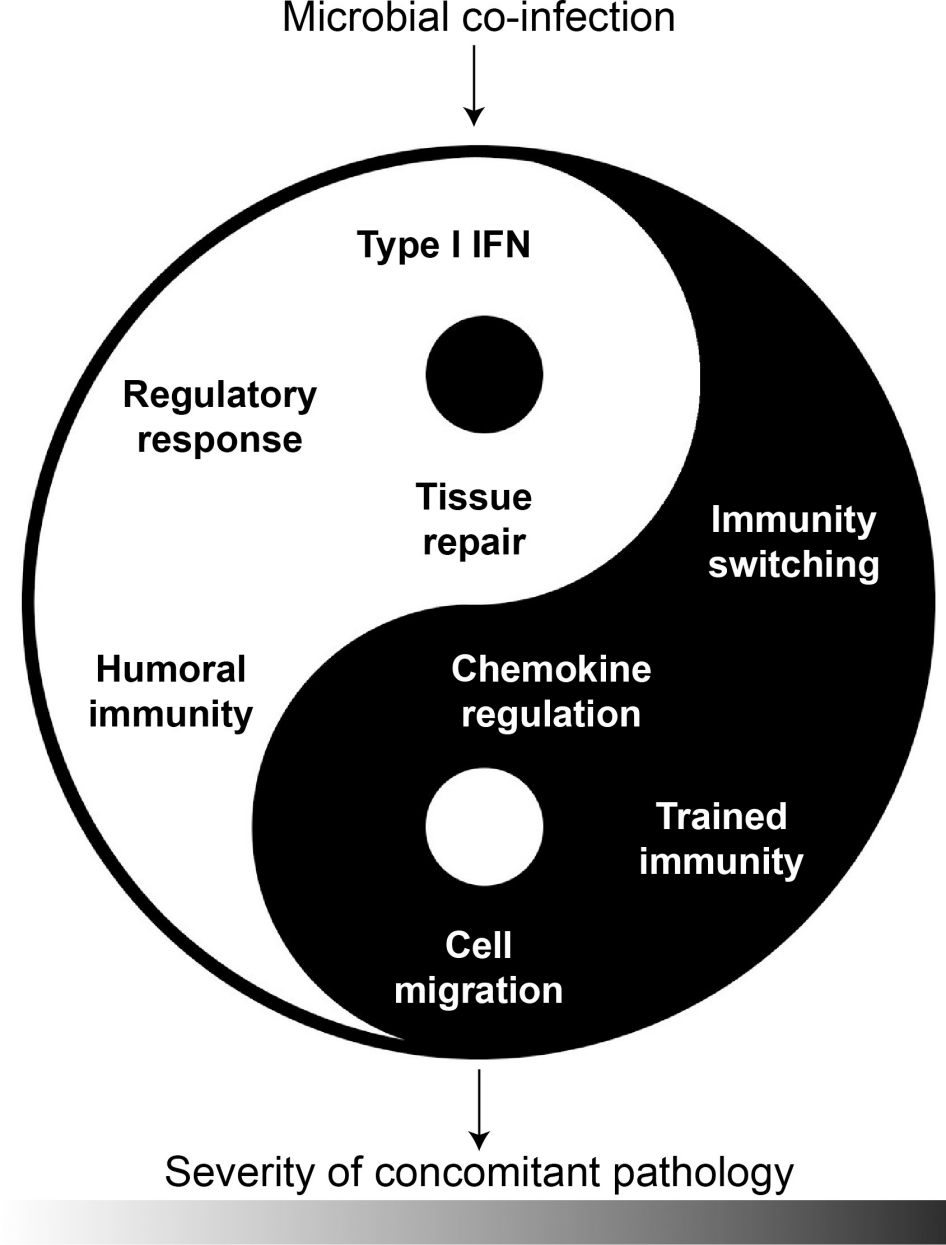
Microbial (co)infection can positively or negatively change the clinical outcome of concomitant infectious and autoimmune diseases through a variety of mechanisms.

It is now well accepted that germ exposure, an element of the global “exposome,” can positively or negatively affect the clinical evolution of concomitant infectious or autoimmune pathologies. Although a causal link remains difficult to establish in humans because of the complexity of intrinsic and extrinsic factors that influence disease progression, experimental studies have demonstrated that a preestablished, simultaneous, or subsequent infection can either ameliorate or exacerbate a concurrent pathology. These studies have revealed the diverse immunological processes by which an infection modulates the clinical outcome of a concomitant disease, although in most cases, the actual effects remain hard to predict. As detailed above and summarized in **Fig 5**, the infection-induced immunomodulation mechanisms include the dual roles of IFN-I, various regulatory pathways and cells, modifications in tissue repair and tolerance to damage, changes in the polarization of immune cells, dysregulated chemoattraction and immune cell migration, and trained immunity.

In the future, more investigations at the cellular, molecular, and genetic levels are needed to characterize microbe-derived molecules that carry these immunomodulatory effects. This would be the basis for the emergence and translational applications of microbial-based therapy. Currently, several clinical trials already aim to use microbes or microbe-derived products for the treatment of different types of infectious and autoimmune diseases (for review [114]). While some results are encouraging, this type of therapy is not yet mature, and more studies about the safety and efficacy are required.

Finally, it is important to highlight that the huge impact of infection on the host immune status has shed light on a weak spot of current vaccine development strategies. When living in areas with high incidence of helminth infection, children display a reduced H1N1-specific antibody response compared to those living in low incidence areas [115], possibly because vaccines that are developed and tested in the Western world may be less efficient in helminth-endemic areas because of the major impact of helminths on the immune system. In an era of renewed interest for large-scale vaccination, such data underscore the necessity of better evaluating the coinfection risks before implementing therapeutic or vaccine strategies in these endemic areas.
